# From the North Sea to Drug Repurposing, the Antiseizure Activity of Halimide and Plinabulin

**DOI:** 10.3390/ph15020247

**Published:** 2022-02-18

**Authors:** Daniëlle Copmans, Sara Kildgaard, Emma Roux, Michèle Partoens, Gert Steurs, Xinhui Wang, Wim M. De Borggraeve, Camila V. Esguerra, Alexander D. Crawford, Thomas O. Larsen, Peter A. M. de Witte

**Affiliations:** 1 Laboratory for Molecular Biodiscovery, Department of Pharmaceutical and Pharmacological Sciences, KU Leuven, Herestraat 49 box 824, 3000 Leuven, Belgium; emma.roux@kuleuven.be (E.R.); michele.partoens@kuleuven.be (M.P.); c.v.esguerra@farmasi.uio.no (C.V.E.); alexander.dettmar.crawford@nmbu.no (A.D.C.); 2 Department of Biotechnology and Biomedicine, Technical University of Denmark, Søltofts Plads, Building 221, 2800 Kgs. Lyngby, Denmark; sarakildgaard@gmail.com (S.K.); xinwang@dtu.dk (X.W.); 3 Molecular Design and Synthesis, Department of Chemistry, KU Leuven, Celestijnenlaan 200f box 2404, 3001 Leuven, Belgium; gert.steurs@kuleuven.be (G.S.); wim.deborggraeve@kuleuven.be (W.M.D.B.)

**Keywords:** marine natural products, drug discovery, epilepsy, zebrafish, plinabulin, halimide

## Abstract

PharmaSea performed large-scale in vivo screening of marine natural product (MNP) extracts, using zebrafish embryos and larvae, to identify compounds with the potential to treat epilepsy. In this study, we report the discovery of two new antiseizure compounds, the 2,5-diketopiperazine halimide and its semi-synthetic analogue, plinabulin. Interestingly, these are both known microtubule destabilizing agents, and plinabulin could have the potential for drug repurposing, as it is already in clinical trials for the prevention of chemotherapy-induced neutropenia and treatment of non-small cell lung cancer. Both halimide and plinabulin were found to have antiseizure activity in the larval zebrafish pentylenetetrazole (PTZ) seizure model via automated locomotor analysis and non-invasive local field potential recordings. The efficacy of plinabulin was further characterized in animal models of drug-resistant seizures, i.e., the larval zebrafish ethyl ketopentenoate (EKP) seizure model and the mouse 6 Hz psychomotor seizure model. Plinabulin was observed to be highly effective against EKP-induced seizures, on the behavioral and electrophysiological level, and showed activity in the mouse model. These data suggest that plinabulin could be of interest for the treatment of drug-resistant seizures. Finally, the investigation of two functional analogues, colchicine and indibulin, which were observed to be inactive against EKP-induced seizures, suggests that microtubule depolymerization does not underpin plinabulin’s antiseizure action.

## 1. Introduction

PharmaSea was an FP7-funded European marine biodiscovery consortium, consisting of 24 partners (non-profit organizations, academia, and SME) from 13 countries, that, from 2012 to 2017, explored oceans in search of novel antibiotic, anti-inflammatory, and neuroactive compounds isolated from marine microorganisms [1,2,3]. The interest to develop drugs of marine origin derives from the fact that marine species can produce unique secondary metabolites in terms of chemistry and bioactivity, as these organisms have evolved to survive under extreme conditions and are largely unexplored given the challenge to access them [1,4]. The search for new microbial diversity by PharmaSea also included extreme marine environments, such as deep ocean trenches and cold and hot vent habitats [1].

This systematic search led to the discovery of many novel molecules and the improved understanding of the therapeutic potential of new and already known small molecules (selected references [2,3,5,6,7,8]). In this paper, we report how zebrafish-based screening of marine natural product (MNP) extracts for novel compounds with the potential to treat epilepsy led to the discovery of the pronounced antiseizure activity of plinabulin, a known drug candidate that is in clinical trials for other therapeutic indications (i.e., the treatment of non-small cell lung cancer (NSCLC) in combination with docetaxel, as well as for the prevention of chemotherapy-induced neutropenia (CIN)) [9,10,11,12,13,14].

The embryonic and larval zebrafish model used is particularly interesting for marine in vivo drug discovery because: (i) it captures the complexity of a whole-body organism and the vertebrate central nervous system; (ii) it can be used for phenotype-based drug discovery that allows the discovery of effective compounds that can have any mode of action; (iii) it can be used for high-throughput screening, as embryos and larvae fit in multi-well plates given their small sizes; and, consequently, (iv) it requires only sub-milligram quantities for hit selection and validation [15,16,17,18]. The final point is especially important for marine drug discovery, as the quantity of MNPs for screening purposes is often scarce.

Epilepsy is one of the most common severe chronic neurological diseases; it affects more than 70 million people worldwide [19,20]. It is characterized by an enduring predisposition to experience unprovoked seizures and involves neurobiological, cognitive, psychological, and social aspects, as well as increased risks for premature death [21]. While over 30 antiseizure drugs are on the market, 35% of all patients continue to suffer from seizures and their devastating consequences due to so-called drug-resistant epilepsy [22,23]. Therefore, the search for novel antiseizure compounds effective against drug-resistant seizures is of utmost importance.

We previously reported on the discovery of the antiseizure activity of the isoquinoline alkaloids TMC-120A and TMC-120B, which were isolated by bioactivity-guided purification from the marine-derived fungus *Aspergillus insuetus* IBT 28443 [3]. The fungus was collected from a seawater trap set in the North Sea, in between Norway and Denmark. TMC-120A and TMC-120B were identified as two out of three active constituents from the hit SK0107, one of the more polar fractions from the initial reversed-phase chromatographic separation of the crude extract of *Aspergillus insuetus* IBT 28443. We here describe the identification and study of halimide, the other active constituent. Halimide is a fungal diketopiperazine metabolite also known as phenylahistin, which was reported to have been isolated from an *Aspergillus ustus* NSC-F038 culture broth in 1997 [24]. The *S*-enantiomer (L-phenylalanine residue) was identified as a novel cell-cycle inhibitor that exhibited antitumor activity in vitro as well as in vivo, due to its microtubule destabilizing effect, by interacting with the colchicine-binding site on tubulin [25,26]. Halimide was of particular interest for antiseizure drug (ASD) discovery and development as its semisynthetic analogue, plinabulin, is currently in phase III clinical trials for the prevention of chemotherapy-induced neutropenia and the treatment of non-small cell lung cancer, in combination with docetaxel [10,11,12]. Thus, plinabulin could have the potential for drug repurposing towards epilepsy. In this study, we report the antiseizure activity of halimide and plinabulin in the zebrafish pentylenetetrazole (PTZ) seizure model, a well-known standard model used for drug discovery within PharmaSea, as well as the characterization of plinabulin’s activity against drug-resistant seizures in both the recently developed larval zebrafish ethyl ketopentenoate (EKP) seizure model [27] and the well-known mouse 6 Hz psychomotor seizure model [28,29]. Finally, two functional analogues of plinabulin, colchicine and indibulin, were also tested in the zebrafish EKP model to investigate whether microtubule depolymerization can explain the antiseizure activity of plinabulin. 

## 2. Results

### 2.1. Marine Antiseizure Drug Discovery and Bioactivity-Guided Purification of Halimide

Over 2000 MNP extracts, including both crude extracts and pre-fractionated extracts, provided by PharmaSea partners, were screened for neuroactivity using the embryonic zebrafish photomotor response (PMR) assay, followed by a secondary screen (of neuroactive hits) for antiseizure activity using the larval zebrafish PTZ seizure model (locomotor assay) [3]. The PMR is a stereotypical behavior of 30–40 h post-fertilization (hpf) zebrafish embryos that is triggered by two subsequent high-intensity light pulses and is used for high-throughput neuroactive drug discovery because of its robustness and behavioral fingerprinting utility [30,31,32]. The zebrafish PTZ seizure model is a fully characterized, well-known model of chemically-induced seizures commonly used for ASD discovery [33,34,35]. The convulsant PTZ (20 mM) is administered to the swimming water of larvae of 7 days post-fertilization (dpf) and induces typical seizure-like behavior that is characterized by high-speed swimming, whirlpool-like circling, clonus-like seizures, and loss of posture [33,34]. All details of the drug discovery process can be found in our previous publication [3]. The behavioral and electrophysiological antiseizure activity of the positive control valproate in the zebrafish PTZ seizure model is shown in the supporting information (Figure S1A,B).

**Figure 1:**
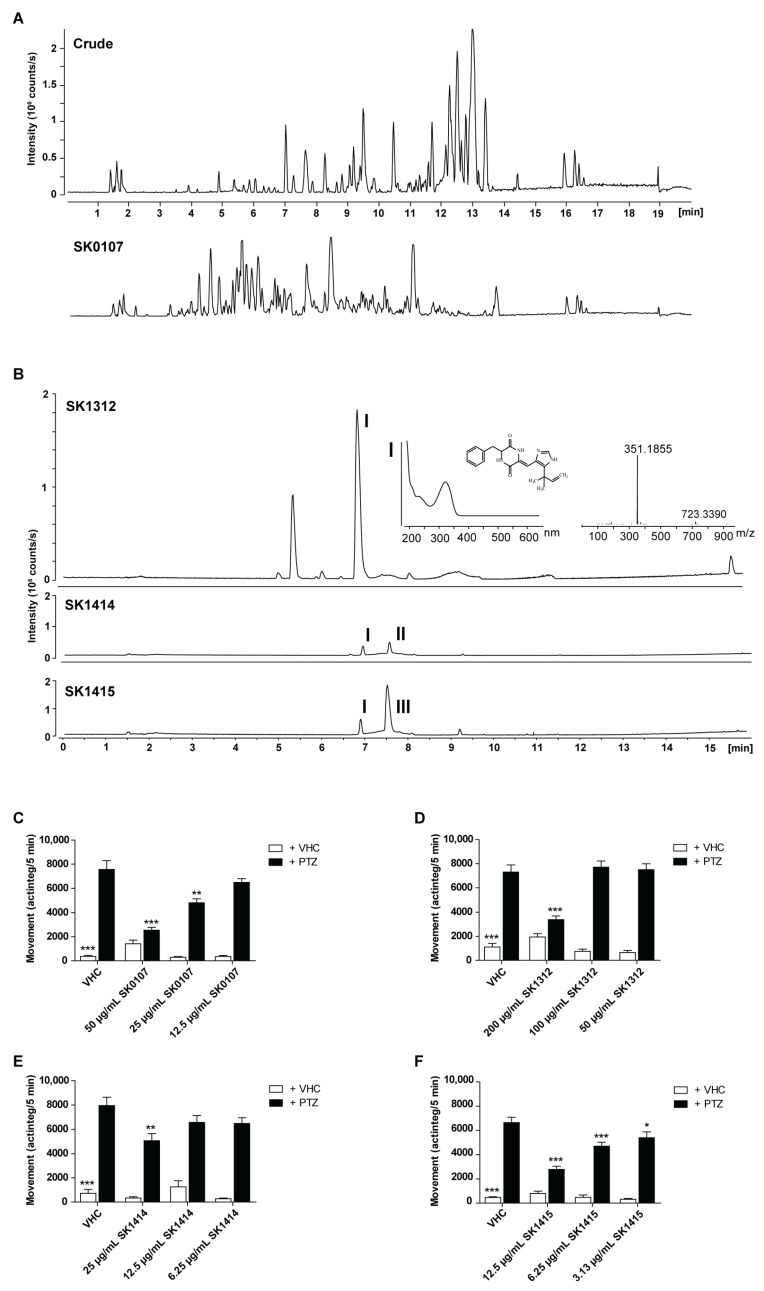
Bioactivity-guided identification of the active compound halimide of antiseizure hit SK0107. (**A**) *Aspergillus insuetus* IBT 28443 cultivated on czapek yeast extract agar (CYA) and yeast extract sucrose agar (YES) media for 9 days at 25 °C in the dark. Base peak chromatograms (BPC) of the crude extract and bioactive fraction SK0107 in positive electrospray ionization mode (ESI+). (**B**) *Aspergillus insuetus* IBT 28443 cultivated on CYA media for 9 days at 25 °C in the dark. ESI+ BPC chromatograms of the most bioactive fraction (SK1312) from first reversed-phase fractionation and of the two most bioactive fractions (SK1414 and SK1415) from the second reversed-phase fractionation. UV/Vis and HRMS spectra shown for halimide (**I**). TMC-120A (**II**) and TMC-120B (**III**) peaks are also indicated. (**C**–**F**) Antiseizure activity of SK0107 (means are pooled from three independent experiments with 12 replicate wells per condition each) (**C**), SK1312 (*n* = 23–24 replicate wells per condition) (**D**), SK1414 (*n* = 10–11 replicate wells per condition) (**E**), and SK1415 (*n* = 22 replicate wells per condition) (**F**) in the zebrafish pentylenetetrazole (PTZ) seizure model after 2 h of treatment. Behavioral data is expressed in mean actinteg units per 5 min (±SEM) during the 30 min recording period. Statistical analysis: (**C**–**F**) one-way ANOVA with Dunnett’s multiple comparison test (GraphPad Prism 5, San Diego, CA, USA). Significance levels: * *p* ≤ 0.05; ** *p* ≤ 0.01; *** *p* ≤ 0.001. Abbreviation: vehicle: VHC.

Among the antiseizure hits was MNP SK0107 (Figure 1A), one of the more polar fractions from the initial reversed-phase chromatographic separation of the crude extract of *Aspergillus insuetus* IBT 28443. SK0107 showed significant and concentration-dependent activity against PTZ-induced seizure behavior, as measured via automated video recording (ZebraBox, ViewPoint, Lyon, France) (Figure 1C).

**Figure 2:**
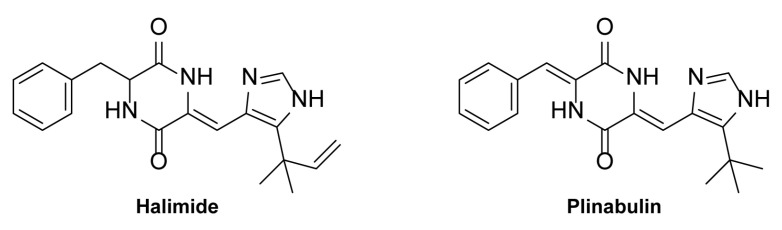
Chemical structures of the 2,5-diketopiperazine halimide and commercially available structural analogue plinabulin.

Bioactivity-guided purification (Figure 1) led to the identification of TMC-120A and TMC-120B, as previously described [3], and halimide, reported in this paper. In brief, a large-scale extract was prepared from the cultivation of *Aspergillus insuetus* IBT 28443 on czapek yeast extract agar (CYA) media for 9 days (in the dark, at 25 °C) and bioactivity-guided purification was performed through several reversed phase purification steps until single-compound isolation. In SK1414 and SK1415, the two most bioactive fractions from the second fractionation of the crude extract, three compounds were tentatively identified by ultra-high performance liquid chromatography-diode array detection-quadrupole time of flight mass spectrometry (UHPLC-DAD-QTOFMS) (Figure 1B,E,F). The two related isoquinoline compounds, TMC-120A and TMC-120B [3], and one compound with the pseudomolecular ion, [M + H]^+^ *m/z* 351.1818 (mass accuracy −0.77 ppm), from which the molecular formula was established to be C_20_H_22_N_4_O_2._ A search in Antibase2012 for the formula revealed a possible candidate, halimide (Figure 2), supported by UV/Vis data consistent with literature and production by related fungal species (*Aspergillus ustus*). The structure of halimide was confirmed by elucidation of the structure by 1D and 2D NMR spectroscopy and the comparison of ^1^H and ^13^C chemical shifts to literature data [24].

To enable further analysis and investigation of halimide, another fungal strain, *Aspergillus ustus* IBT 4133, was chosen for isolation of halimide in greater amounts (>15 mg). This strain was found to produce halimide as its main compound in a previous investigation of crude extracts on CYA and yeast extract sucrose agar (YES) of various closely related species belonging to the *Aspergillus* section *Usti* [3]. The structural semisynthetic analogue plinabulin (Figure 2) was commercially available and therefore purchased to investigate its antiseizure activity.

### 2.2. Halimide Ameliorates Seizures and Epileptiform Brain Activity in the Zebrafish PTZ Seizure Model

**Figure 3:**
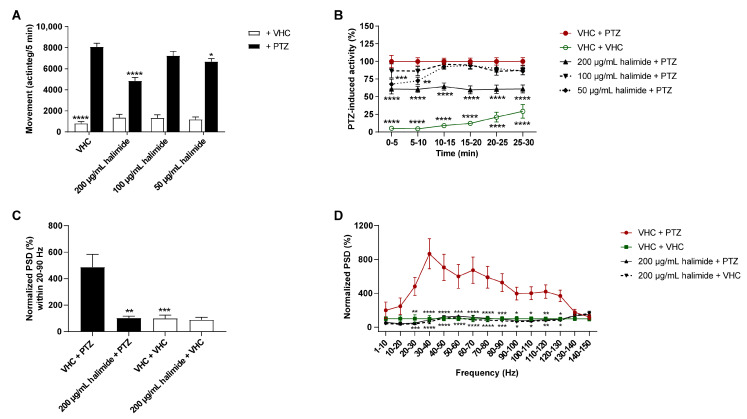
Antiseizure and anti-epileptiform analysis of halimide in the zebrafish PTZ seizure model. Antiseizure activity (**A**,**B**) and anti-epileptiform activity (**C**,**D**) of halimide in the zebrafish pentylenetetrazole (PTZ) seizure model after 2 h of incubation. (**A**,**B**) Behavioral data is expressed in mean actinteg units per 5 min (±SEM) during the 30 min recording period (**A**) and over consecutive time intervals (**B**). Data is normalized against the vehicle (VHC) + PTZ control condition (**B**). (**C**,**D**) PTZ-induced epileptiform brain activity and the anti-epileptiform effect of 200 µg/mL halimide recorded via non-invasive local field potential recordings. Electrophysiological data is normalized against the VHC + VHC control condition and expressed as normalized power spectral density (PSD) (mean ± SEM) per larva within the 20–90 Hz region (**C**) and as normalized PSD (mean ± SEM) per larva per 10 Hz frequency band from 1–150 Hz (**D**). (**A**,**B**) Data are pooled from five independent experiments with each 6–11 replicate wells per condition: VHC + PTZ (*n* = 53), compound + PTZ (*n* = 52–53), VHC + VHC (*n* = 53), and compound + VHC (*n* = 41). (**C**,**D**) Number of replicates per condition: VHC + PTZ (*n* = 17), VHC + VHC (*n* = 13), halimide + PTZ (*n* = 11) and halimide + VHC (*n* = 12). Statistical analysis: (**A**,**C**) one-way ANOVA with Dunnett’s multiple comparison test, (**B**,**D**) two-way ANOVA with Dunnett’s multiple comparison test, (**C**,**D**) outliers were removed via the ROUT test (Q = 1%) (GraphPad Prism 9, San Diego, CA, USA). Significance levels: * *p* ≤ 0.05; ** *p* ≤ 0.01; *** *p* ≤ 0.001; **** *p* ≤ 0.0001.

To confirm that halimide isolated from the most bioactive fractions was indeed an active constituent, its antiseizure activity was investigated in the zebrafish PTZ seizure model (Figure 3A,B). Larvae were treated with either vehicle (VHC) or halimide (using the maximum tolerated concentration (MTC), MTC/2, and MTC/4) for 2 h. Halimide significantly lowered PTZ-induced seizure behavior at its MTC (*p* ≤ 0.0001, Figure 3A) and MTC/4 (*p* ≤ 0.05, Figure 3A) during the 30 min recording period, these results are comparable to the positive control valproate (Figure S1A). A more detailed analysis of the behavior over consecutive 5 min time intervals revealed a significant reduction of PTZ-induced seizure behavior during the entire time period (*p* ≤ 0.0001, Figure 3B). These data demonstrate the antiseizure activity of halimide and confirm that the isolated compound is indeed an active constituent of the antiseizure hit SK0107 and the bioactive fractions SK1312, SK1414, and SK1415. The higher antiseizure efficacy of hit SK0107 in comparison with halimide is possibly due to a synergistic action with the other active constituents identified, i.e., TMC-120A and TMC-120B [3].

To determine whether halimide affects the PTZ-induced hyperexcitable state of the brain characterized by epileptiform discharges [36] in addition to influencing behavioral antiseizure activity, local field potential (LFP) recordings [37] were non-invasively measured from the midbrain (optic tectum) of zebrafish larvae (Figure 3C,D and Figure S2). Larvae were treated with either VHC or 200 µg/mL halimide for 2 h followed by a 15 min exposure to PTZ or VHC prior to LFP measurements. Pre-exposure to PTZ resulted in a significant increase of epileptiform electrical discharges compared to VHC. Instead of visual analysis of the LFP recordings, which is time-consuming and prone to the personal interpretation of the investigator, power spectral density (PSD) computation was performed, which is an automated, fast, and objective alternative analysis method [35]. The PSD estimates of electrical discharges in each LFP recording were determined per 10 Hz frequency band, ranging from 0 to 150 Hz using Welch’s method, and normalized against the VHC control (Figure 3D). Larvae treated with PTZ (VHC + PTZ) showed a significant elevation in PSD (*p* ≤ 0.05, *p* ≤ 0.01, *p* ≤ 0.001, *p* ≤ 0.0001) compared with VHC-treated larvae (VHC + VHC) within the 20–130 Hz region. The highest differences between the PTZ and VHC controls were observed between 20–90 Hz, which was selected as the region of interest. Similar to the positive control, valproate (Figure S1B), halimide showed significant and pronounced anti-epileptiform activity, lowering PTZ-induced elevated PSD of PTZ-treated larvae (halimide + PTZ) to a level comparable to VHC-treated controls (VHC + VHC) within the 20–90 Hz region (*p* ≤ 0.01, Figure 3C) and per the 10 Hz frequency bands within the 20–130 Hz region (*p* ≤ 0.05, *p* ≤ 0.01, *p* ≤ 0.001, and *p* ≤ 0.0001; Figure 3D). Thus, these data show that halimide counteracts the hyperexcitable state of the brain. Of note, halimide treatment in the absence of PTZ did not affect normal swimming behavior (Figure 3A) or brain activity (Figure 3C,D).

### 2.3. The S-Enantiomer of Halimide Is Active within the Scalemic Mixture

**Figure 4:**
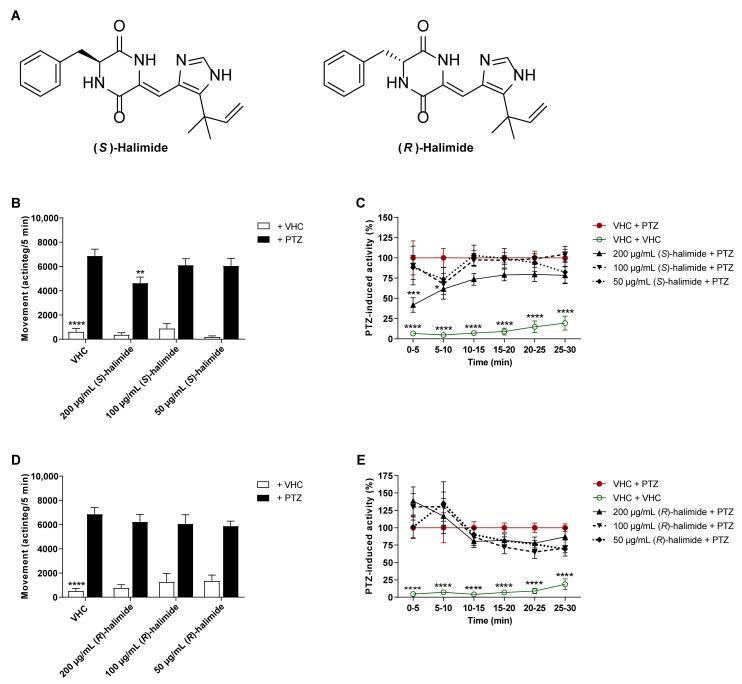
Behavioral antiseizure analysis of the *R*- and *S*-enantiomers of halimide in the zebrafish PTZ seizure model. (**A**) Chemical structures of (*S*)-halimide and (*R*)-halimide. (**B**–**E**) Antiseizure activity of (*S*)-halimide (**B**,**C**) and (*R*)-halimide (**D**,**E**) in the zebrafish pentylenetetrazole (PTZ) seizure model after 2 h of incubation. Behavioral data is expressed in mean actinteg units per 5 min (±SEM) during the 30 min recording period (**B**,**D**) and over consecutive time intervals (**C**,**E**). Data is normalized against the vehicle (VHC) + PTZ control condition (**C**,**E**). Data are pooled from two independent experiments with a total of 20 replicate wells for VHC + VHC, VHC + PTZ, and compound + PTZ conditions, and 11–12 replicate wells for compound + VHC conditions. Statistical analysis: (**B**,**D**) one-way ANOVA with Dunnett’s multiple comparison test, (**C**,**E**) two-way ANOVA with Dunnett’s multiple comparison test (GraphPad Prism 9, San Diego, CA, USA). Significance levels: * *p* ≤ 0.05; ** *p* ≤ 0.01; *** *p* ≤ 0.001; **** *p* ≤ 0.0001.

In this study, halimide was discovered as a scalemic mixture of the *R*- and *S*-enantiomer, based on the measurement of the optical rotation and Marfey’s analysis, which suggested a ratio of approximately 3:1 of the D- and L-phenylalanine residue. This is consistent with prior literature [24]. Separation of the two compounds was therefore performed by chiral HPLC, supported by measurement of the optical rotations and comparisons to literature [24], to enable the evaluation of each enantiomer individually. The antiseizure activity of the enantiomers was tested in the zebrafish PTZ seizure model after 2 h of treatment (Figure 4). (*S*)-Halimide significantly lowered PTZ-induced seizure behavior at 200 µg/mL (*p* ≤ 0.01, Figure 4B) during the 30 min recording period. A more detailed analysis of the behavior over consecutive 5 min time intervals revealed a significant reduction of PTZ-induced seizure behavior during the first 10 min (*p* ≤ 0.001 and *p* ≤ 0.05, Figure 4C) of recording. (*R*)-Halimide displayed no significant effect (Figure 4D,E). These data reveal that the activity of the scalemic mixture of halimide (3:1 *R*:*S*), as observed in Figure 3, is due to the *S*-enantiomer of halimide.

### 2.4. Plinabulin Ameliorates Seizures and Epileptiform Brain Activity in the Zebrafish PTZ Seizure Model

**Figure 5:**
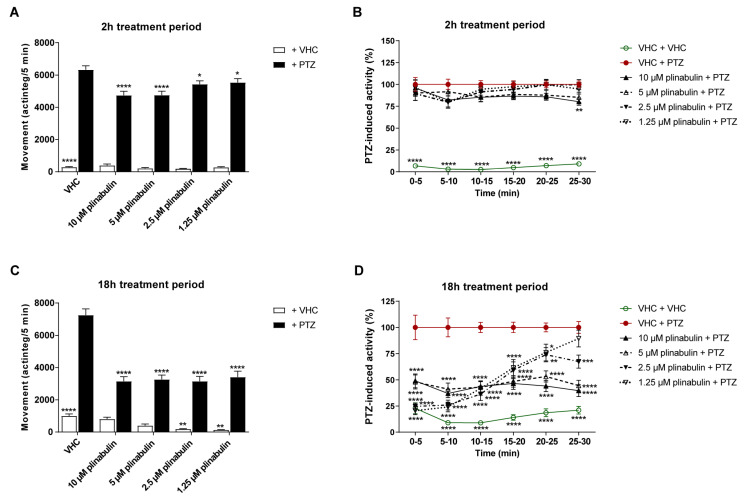
Behavioral antiseizure analysis of plinabulin after a short- and long-term treatment in the zebrafish PTZ seizure model. Antiseizure activity of plinabulin in the zebrafish pentylenetetrazole (PTZ) seizure model after a treatment period of 2 h (**A**,**B**) and 18 h (**C**,**D**). Behavioral data is expressed in mean actinteg units per 5 min (±SEM) during the 30 min recording period (**A**,**C**) and over consecutive 5 min time intervals (**B**,**D**). Data is normalized against the vehicle (VHC) + PTZ control condition (**B**,**D**). Data were pooled from ten independent experiments with 10 replicate wells per test condition (*n* = 100 for VHC + VHC and VHC + PTZ and *n* = 70 for plinabulin + VHC and plinabulin + PTZ) (**A**,**B**), and from three independent experiments with 10 replicate wells per test condition (*n* = 30) (**C**,**D**). Statistical analysis: one-way ANOVA with Dunnett’s multiple comparison test (**A**,**C**), two-way ANOVA with Dunnett’s multiple comparison test (**B**,**D**) (GraphPad Prism 9, San Diego, CA, USA). Significance levels: * *p* ≤ 0.05; ** *p* ≤ 0.01; *** *p* ≤ 0.001; **** *p* ≤ 0.0001.

Plinabulin (Figure 2), the commercially available structural analogue of halimide, was tested in the zebrafish PTZ seizure model after both 2 and 18 h of treatment to examine whether this 2,5-diketopiperazine also shows antiseizure activity. Given plinabulin’s limited water solubility [38,39], the highest test concentration was determined not by its MTC but by its maximum soluble concentration in embryo medium (1% DMSO), which was 10 µM. After 2 h of exposure, significant antiseizure activity was observed at all concentrations tested (*p* ≤ 0.0001 and *p* ≤ 0.05, Figure 5A), though the efficacy was rather limited. After 18 h of exposure, plinabulin was clearly more effective, significantly reducing PTZ-induced seizure behavior at all concentrations tested (i.e., 1.25–10 µM) during the 30 min recording interval (*p* ≤ 0.0001) (Figure 5C). It was thus found to be more effective than the positive control, valproate (Figure S1A). A more detailed analysis of the 30 min recording over consecutive 5 min intervals revealed the activity profiles of plinabulin as a function of time and concentration (Figure 5D). Of note, after a long-term exposure to plinabulin in the absence of PTZ, zebrafish larvae had significant lower swimming behavior at 1.25 and 2.5 µM (*p* ≤ 0.01), but not at 5 and 10 µM (Figure 5C).

**Figure 6:**
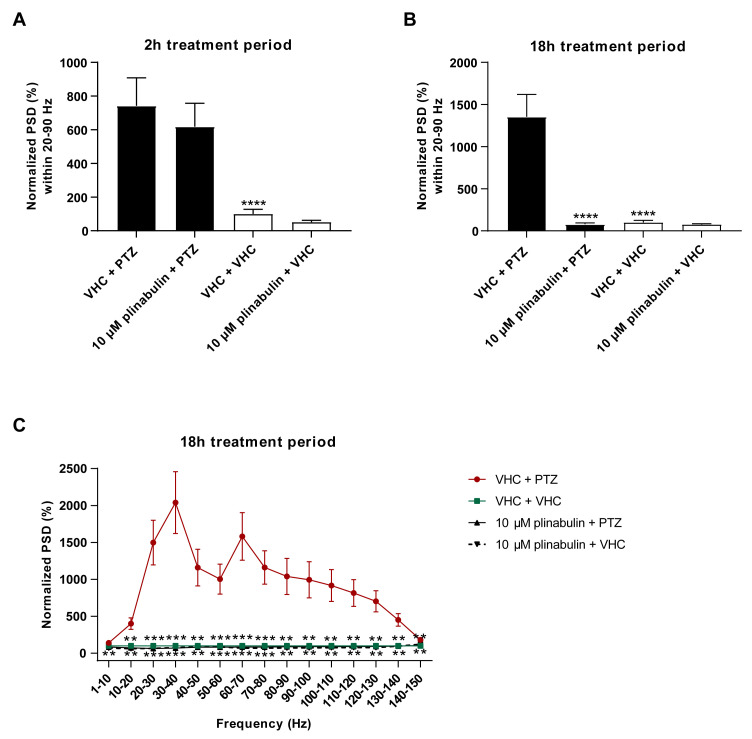
Electrophysiological antiseizure analysis of plinabulin in the zebrafish PTZ seizure model after a short- and long-term treatment. Anti-epileptiform activity of plinabulin after 2 h (**A**) and 18 h (**B**,**C**) in the zebrafish pentylenetetrazole (PTZ) seizure model. Electrophysiological data is normalized against the vehicle (VHC) + VHC control condition and expressed as normalized power spectral density (PSD) (mean ± SEM) per larva within the 20–90 Hz region (**A**,**B**) and as normalized PSD (mean ± SEM) per larva per 10 Hz frequency band from 1–150 Hz (**C**). Number of replicates per test condition was *n* = 12–17. Statistical analysis: one-way ANOVA with Dunnett’s multiple comparison test (**A**,**B**), two-way ANOVA with Dunnett’s multiple comparison test (**C**), outliers were removed via the ROUT test (Q = 1%) (GraphPad Prism 9, San Diego, CA, USA). Significance levels: * *p* ≤ 0.05; ** *p* ≤ 0.01; *** *p* ≤ 0.001; **** *p* ≤ 0.0001.

Next, non-invasive LFP recordings were measured from the optic tectum of zebrafish larvae after short-term (2 h) and long-term (18 h) treatment with 10 µM plinabulin to understand whether plinabulin also counteracts the PTZ-induced hyperexcitable state of the brain, such as halimide. While only a non-significant reduction in PTZ-induced PSD elevation was observed after 2 h pre-exposure (Figure 6A), a complete rescue was observed after 18 h (*p* ≤ 0.0001 (Figure 6B), *p* ≤ 0.001 and *p* ≤ 0.01 (Figure 6C)), in line with the locomotor results and comparable to the efficacy seen for the positive control, valproate (Figures S1B and S2). These data demonstrate that, like halimide, plinabulin has pronounced anti-epileptiform activity. 

### 2.5. Plinabulin Ameliorates Seizures and Epileptiform Brain Activity in the Zebrafish EKP Seizure Model

One of the most important treatment gaps for epilepsy patients is the lack of ASDs that can achieve seizure freedom in patients that suffer from pharmacoresistant epilepsy. Therefore, ASD discovery and development efforts are currently focused on new chemical entities that are active against drug-resistant seizures [23]. Hence, to further characterize the potential of plinabulin as an antiseizure compound, its antiseizure activity was also tested in the larval zebrafish EKP model of drug-resistant seizures [27]. This animal model has recently been developed by our group and, after in-depth pharmacological characterization, was reported to have the potential to select innovative antiseizure compounds. EKP is a lipid-permeable inhibitor of glutamic acid decarboxylase (GAD) that converts glutamate into γ-aminobutyric acid (GABA) [27]. GAD is a key enzyme in the dynamic regulation of neural network excitability [27]. Importantly, the decrease of GAD activity in zebrafish is clinically relevant, as lowered GAD activity is associated with several forms of epilepsy which are often pharmacoresistant [40,41,42,43,44]. The behavioral and electrophysiological antiseizure activity of the positive control perampanel in the zebrafish EKP seizure model is shown in the supporting information (Figure S1C,D).

**Figure 7:**
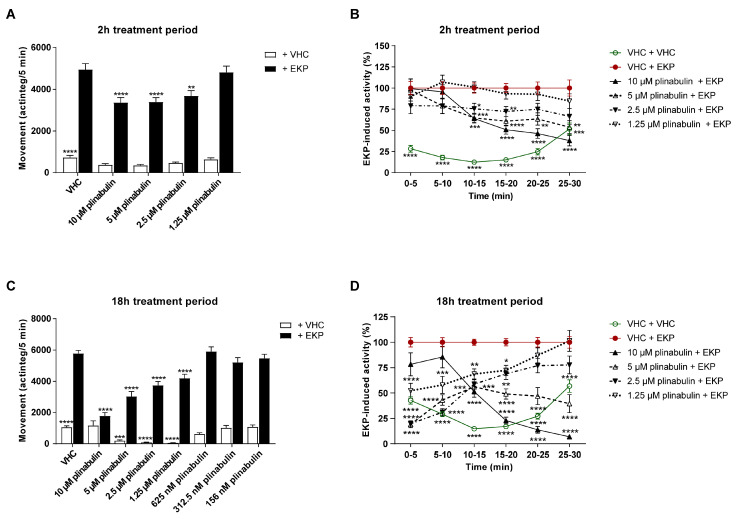
Behavioral antiseizure analysis of plinabulin after short- and long-term treatment in the zebrafish EKP seizure model. Antiseizure activity of plinabulin in the zebrafish ethyl ketopentenoate (EKP) seizure model after a treatment of 2 h (**A**,**B**) and after a treatment of 18 h (**C**,**D**). Behavioral data is expressed in mean actinteg units per 5 min intervals (±SEM) during the 10–25 min recording period (**A**,**C**) and over consecutive 5 min time intervals during the entire 30 min recording period (**B**,**D**). Data is normalized against the vehicle (VHC) + EKP control condition (**B**,**D**). Data were pooled from four independent experiments with 10 replicate wells per test condition (*n* = 40) (**A**,**B**) and from three or four independent experiments with 10 replicate wells per test condition (*n* = 70 for VHC + VHC and VHC + EKP, *n* = 28–30 for 5–10 µM plinabulin conditions, and *n* = 39–40 for 0.156–2.5 µM plinabulin conditions) (**C**,**D**). Statistical analysis: one-way ANOVA with Dunnett’s multiple comparison test (**A**,**C**), two-way ANOVA with Dunnett’s multiple comparison test (**B**,**D**) (GraphPad Prism 9, San Diego, CA, USA). Significance levels: * *p* ≤ 0.05; ** *p* ≤ 0.01; *** *p* ≤ 0.001; **** *p* ≤ 0.0001.

The antiseizure activity of plinabulin was investigated after both 2 h and 18 h periods of exposure. After the short-term treatment of 2 h, plinabulin significantly and concentration-dependently lowered EKP-induced seizure behavior at 10 µM (*p* ≤ 0.0001), 5 µM (*p* ≤ 0.0001), and 2.5 µM (*p* ≤ 0.01) during the 10–25 min recording period (Figure 7A). This period was selected from the 30 min recording, because EKP-induced seizure behavior was observed to be most pronounced during this time window. This behavioral trend is also clear from the more detailed analysis of the 30 min recording period in consecutive 5 min intervals (Figure 7B). After the long-term treatment of 18 h, plinabulin significantly and concentration-dependently lowered the EKP-induced seizure behavior at 1.25–10 µM during the 10–25 min recording period (*p* ≤ 0.0001, Figure 7C). The more detailed analysis of the 30 min recording period in consecutive 5 min intervals reveals that plinabulin is also active outside the 10–25 min window (Figure 7D). Again, the efficacy of plinabulin was more pronounced after the 18 h treatment compared to the 2 h treatment per and comparable to that of the positive control, perampanel (Figure S1C). However, after 18 h of exposure, the normal swimming behavior in the absence of EKP was also significantly lowered by 1.25–5 µM plinabulin (*p* ≤ 0.001 and *p* ≤ 0.0001), but not at higher or lower concentrations (Figure 7C).

**Figure 8:**
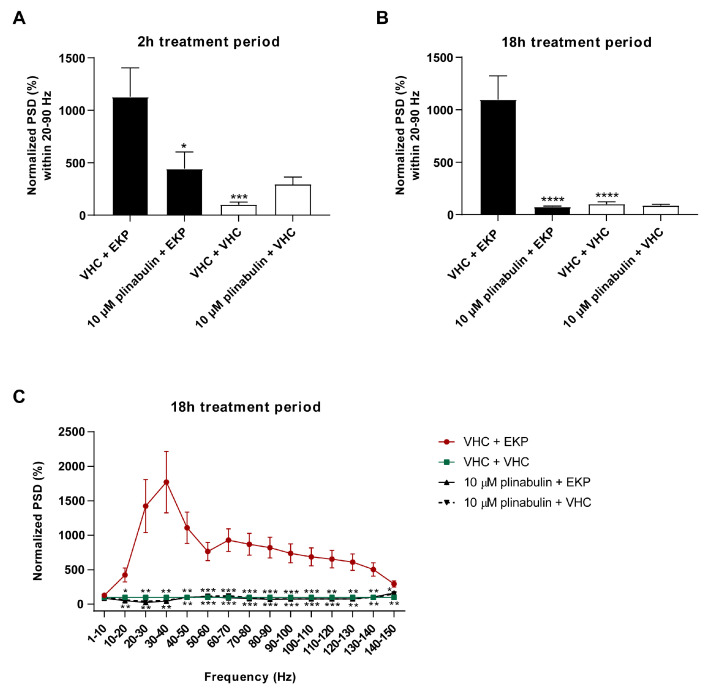
Electrophysiological antiseizure analysis of plinabulin in the zebrafish EKP seizure model after a short- and long-term treatment. Anti-epileptiform activity of plinabulin after 2 h (**A**) and 18 h (**B**,**C**) in the zebrafish ethyl ketopentenoate (EKP) seizure model. Electrophysiological data is normalized against the vehicle (VHC) + VHC control condition and expressed as normalized power spectral density (PSD) (mean ± SEM) per larva within the 20–90 Hz region (**A**,**B**) and as normalized PSD (mean ± SEM) per larva per 10 Hz frequency band from 1–150 Hz (**C**). Number of replicates per test condition was *n* = 13–16. Statistical analysis: one-way ANOVA with Dunnett’s multiple comparison test (**A**,**B**), two-way ANOVA with Dunnett’s multiple comparison test (**C**), outliers were removed via the ROUT test (Q = 1%) (GraphPad Prism 9, San Diego, CA, USA). Significance levels: * *p* ≤ 0.05; ** *p* ≤ 0.01; *** *p* ≤ 0.001; **** *p* ≤ 0.0001.

Next, LFP recordings were non-invasively measured from the optic tectum of zebrafish larvae after both a short-term (2 h) and long-term (18 h) treatment with 10 µM plinabulin to understand whether plinabulin also counteracts the EKP-induced hyperexcitable state of the brain. A significant reduction (*p* ≤ 0.05) in EKP-induced PSD elevation was observed after 2 h pre-exposure (Figure 8A), and a complete rescue was observed after 18 h (*p* ≤ 0.0001 (Figure 8B), *p* ≤ 0.001 and *p* ≤ 0.01 (Figure 8C)), in line with the locomotor results and comparable to the efficacy seen for the positive control, perampanel (Figures S1D and S3). Thus, plinabulin also shows pronounced anti-epileptiform activity in the zebrafish EKP model.

### 2.6. Microtubule Destabilization Is Insufficient for the Antiseizure Mechanism of Action of Plinabulin

**Figure 9:**
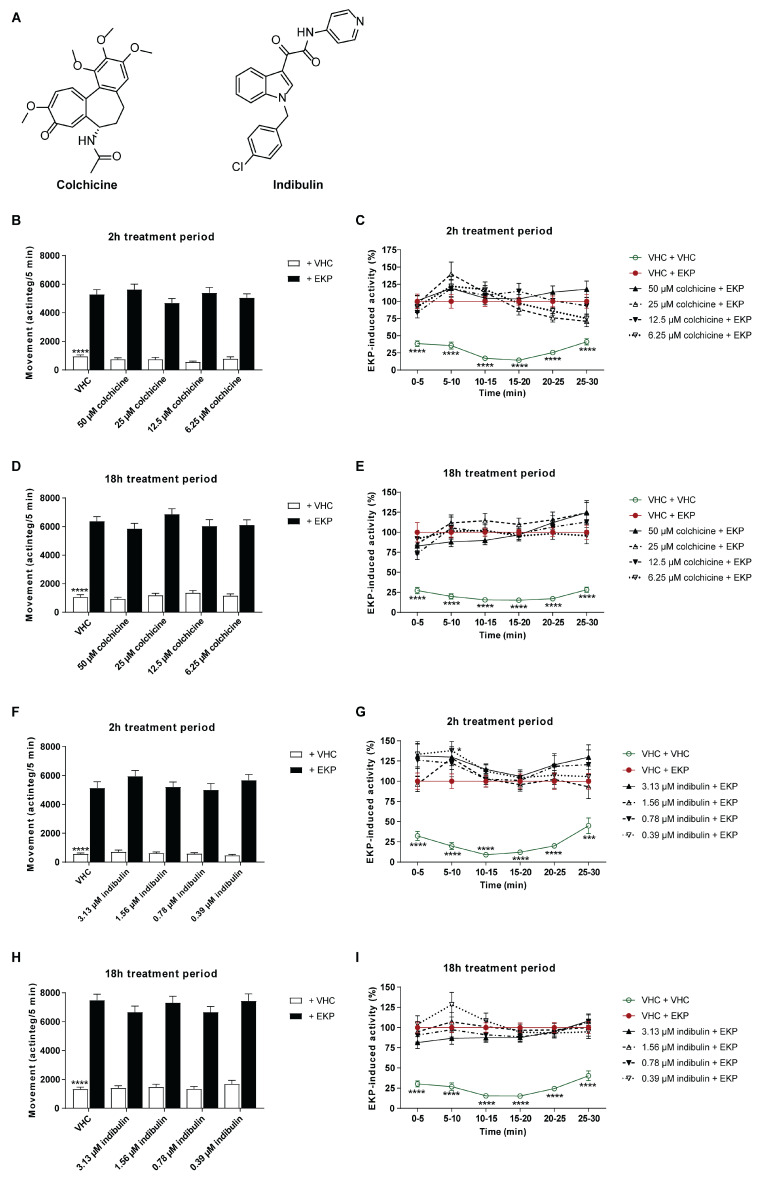
Behavioral antiseizure analysis of colchicine and indibulin in the zebrafish EKP seizure model after short- and long-term treatment. (**A**) Chemical structures of colchicine and indibulin. (**B**–**I**) Antiseizure activity of colchicine (**B**–**E**) and indibulin (**F**–**I**) after a treatment of 2 h (**B**,**C**,**F**,**G**) and after a treatment of 18 h (**D**,**E**,**H**,**I**) in the zebrafish ethyl ketopentenoate (EKP) seizure model. Behavioral data is expressed in mean actinteg units per 5 min (±SEM) during the 10–25 min recording period (**B**,**D**,**F**,**H**), and over consecutive 5 min time intervals during the entire 30 min recording period (**C**,**E**,**G**,**I**). Data are normalized against the vehicle (VHC) + EKP control condition (**C**,**E**,**G**,**I**). Data were pooled from three independent experiments with 10 replicate wells per test condition (*n* = 30) (**B**–**I**). Statistical analysis: one-way ANOVA with Dunnett’s multiple comparison test (**B**,**D**,**F**,**H**), two-way ANOVA with Dunnett’s multiple comparison test (**C**,**E**,**G**,**I**) (GraphPad Prism 9, San Diego, CA, USA). Significance levels: * *p* ≤ 0.05; ** *p* ≤ 0.01; *** *p* ≤ 0.001; **** *p* ≤ 0.0001.

Plinabulin is currently being tested in clinical trials for different therapeutic indications in the field of cancer therapy [45,46]. Its mode of action has been shown to rely on the binding to β-tubulin, preventing the polymerization of tubulin [13,45,47]. To explore whether a similar mode of action could be responsible for its antiseizure activity, two additional microtubule destabilizing agents, colchicine and indibulin (Figure 9A), were tested in the larval zebrafish EKP seizure model to investigate if these functional analogues also possess antiseizure activity. Colchicine is used in clinical practices for the indication of gout [48], and indibulin is an anticancer agent used against advanced solid tumors [49].

Colchicine’s MTC was determined to be 50 µM and was tested in a concentration range of 6.25–50 µM (Figure 9B–E). Indibulin was tested in a concentration range of 0.39–3.13 µM, where 3.13 µM was the highest soluble concentration in embryo medium (1% DMSO) (Figure 9F–I). Indibulin is characterized by low water solubility [50] and consequently tested at the maximum soluble concentration instead of the MTC.

Neither after 2 h of exposure, nor after 18 h, did the functional analogues show activity against EKP-induced seizure behavior compared to VHC-treated zebrafish larvae (*p* > 0.05, Figure 9). The lack of antiseizure activity of colchicine and indibulin suggests that microtubule depolymerization is not likely to be involved in plinabulin’s antiseizure mode of action.

### 2.7. Plinabulin Ameliorates Seizures in the Mouse 6 Hz Psychomotor Seizure Model

Finally, we investigated whether the antiseizure action of plinabulin observed in the larval zebrafish model translated to a standard rodent seizure model. To that end, we used the mouse 6 Hz (44 mA) psychomotor seizure model, a gold standard in current ASD discovery efforts that is useful for screening and can detect compounds with novel antiseizure mechanisms and with potential against drug-resistant seizures [28,29,51]. It is an acute model of drug-resistant focal impaired awareness seizures [52], previously referred to as complex partial or psychomotor seizures [53], that are induced by a low-frequency, long-duration corneal electrical stimulation [29].

**Figure 10:**
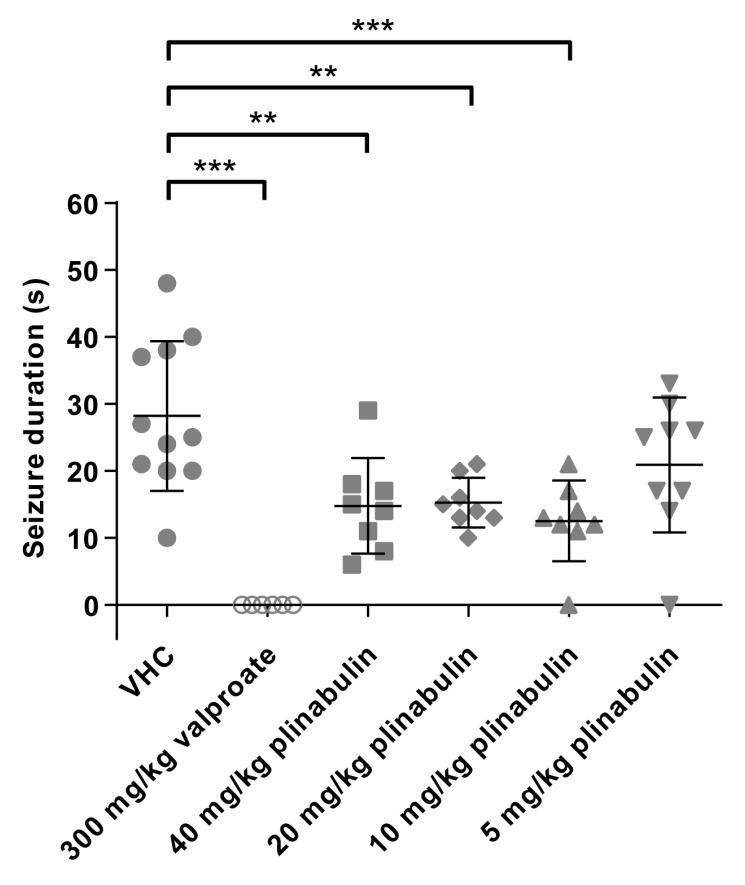
Antiseizure activity analysis of plinabulin in the mouse 6 Hz psychomotor seizure model. Drug-resistant psychomotor seizures were induced by electrical stimulation (6 Hz, 0.2 ms rectangular pulse width, 3 s duration, 44 mA) through the cornea, 30 min after intraperitoneal injection of vehicle (VHC, *n* = 11), positive control valproate (*n* = 6), or plinabulin (*n* = 8–9). Mean seizure durations (±SD) are depicted. Statistical analysis: one-way ANOVA with Dunnett’s multiple comparison test (GraphPad Prism 9). Significance levels: * *p* ≤ 0.05; ** *p* ≤ 0.01; *** *p* ≤ 0.001.

Male NMRI mice were intraperitoneally (i.p.) injected with a 50 µL volume (adjusted to the individual weight) of VHC (DMSO:PEG200 1:1), positive control valproate (300 mg/kg), or plinabulin (5, 10, 20, or 40 mg/kg) 30 min before electrical stimulation (Figure 10). VHC-injected mice showed characteristic seizure behavior with a mean (±SD) duration of 28 s (±11 s). In line with previous studies, mice that were injected with valproate were fully protected against the induced seizures [28,54], as none of the mice showed any seizure after electrical stimulation (*p* ≤ 0.001). Mice i.p. injected with plinabulin had a shorter seizure duration than the VHC control group, which was significant at 40 mg/kg (*p* ≤ 0.01, mean duration of 15 s (±7 s)), 20 mg/kg (*p* ≤ 0.01, mean duration of 15 s (±4 s)), and 10 mg/kg (*p* ≤ 0.001, mean duration of 12.5 s (±6 s)), but not at 5 mg/kg (mean duration of 21 s (±10 s)). Thus, the antiseizure activity of plinabulin that was observed in the larval zebrafish PTZ and EKP seizure models was observed to translate to a standard mouse model of drug-resistant focal seizures.

## 3. Discussion

The interest in pharmacologically active marine natural products as a source for novel drug candidates has increased strongly over time [55,56,57]. The global marine pharmaceutical clinical pipeline in 2020 consisted of 14 marine drugs and 23 drug candidates in phase I–III trials (Clinical Pipeline (midwestern.edu)), including four small molecules in phase III, three of them as an anticancer therapy (plinabulin, lurbinectedin, and salinosporamide A) and one as an analgesic compound (tetrodoxin) [55,56,57,58,59,60]. Despite the value of MNPs and their contribution to the drug development pipeline, there are obvious challenges to the discovery of compounds from a marine source in terms of accessibility, material supply, structural complexity, and the identification of novel chemistry and bioactivity. Advanced techniques for deep-water sampling, automated extraction, pre-fractionation, and rapid dereplication, as well as metagenomics, genome mining, and high-content screening are therefore in use and under constant improvement [57,58,61]. The use of zebrafish embryos and larvae, a small vertebrate animal model, allows medium-throughput in vivo assessment of bioactivities using only microgram quantities [62]. This is a key advantage for (marine) natural product discovery and research, where material is often scarce [62,63] and was, therefore, used by PharmaSea for neuroactive drug discovery [1,2,3]. Other examples of zebrafish-based screening for active marine natural products can be found in [64,65].

Our systematic search for novel antiseizure compounds present in MNP extracts ultimately led to the investigation of the antiseizure properties of plinabulin, a commercially available compound, well-known in the field of cancer therapy—as a drug candidate for the treatment of NSCLC in combination with docetaxel and for the prevention of CIN [9,10,11,12,13,14]. Halimide was identified as an active constituent of the antiseizure hit SK0107, one of the more polar fractions derived from the crude extract of *Aspergillus insuetus* IBT 28443, collected from a seawater trap set in the North Sea. Its antiseizure and anti-epileptiform activities were characterized in the zebrafish PTZ seizure model via locomotor analysis and LFP recordings. While halimide is known to possess antitumor activity [25,26], it has never before been described to be active against epileptic seizures. In fact, to the best of our knowledge, it is the first 2,5-diketopiperazine reported to have antiseizure activity. Of note, 2,6-diketopiperazine derivatives have previously been described with anticonvulsant activity [66,67]. A wide spectrum of biological properties has been reported for 2,5-diketopiperazines, including antitumoral, antiviral, antifungal, antibacterial, antihyperglycemic activities, neuroprotective, and nootropic activity [68]. Of particular interest is the neuroprotective and nootropic action. The 2,5-diketopiperazines structurally related to the thyrotropin-releasing hormone have been reported to prevent or reduce both necrotic and apoptotic cell death in different in vitro models and to reduce lesion volumes and improve cognitive and motor outcomes in rodent models of traumatic brain injury [68,69,70,71]. They have, therefore, been suggested for the treatment of neurodegenerative disorders such as Alzheimer’s disease and Parkinson’s disease [68].

Consistent with prior literature [24], halimide was discovered as a scalemic mixture (3:1 *R*:*S*). Each of the separated enantiomers was tested in the zebrafish PTZ seizure model and indicated the *S*-enantiomer of halimide as the active component. Kanoh and colleagues showed, in line with this, that the *S*-enantiomer was more active than the *R*-enantiomer, exhibiting 33–100-fold more potent antitumor activity in tumor cell lines [25].

Plinabulin, the semisynthetic structural analogue of halimide, also showed pronounced antiseizure and anti-epileptiform activity in the zebrafish PTZ seizure model. Like halimide, plinabulin is shown for the first time to be active against epileptic seizures. Moreover, it also showed pronounced antiseizure and anti-epileptiform activity in the zebrafish EKP model of drug-resistant seizures and was active in the mouse 6 Hz psychomotor seizure model, an acute model of drug-resistant focal impaired awareness seizures that is commonly used for ASD discovery [28,29]. These data demonstrate plinabulin’s effectiveness against drug-resistant seizures, which makes it of particular interest for further investigation within the field of epilepsy.

In both zebrafish models, plinabulin was observed to be more effective after a long-term 18 h treatment compared to a short-term 2 h treatment. This might be explained by an increased compound uptake and/or brain concentration. Given plinabulin’s low water solubility [38,39] and the use of water immersion for compound administration (oral uptake and passive diffusion via skin and gills) to the larvae, a low bioavailability after a short exposure time seems plausible, but currently no pharmacokinetic data are present to support this. Plinabulin’s poor pharmacokinetic properties in general, and low water solubility in particular, have been reported to limit its pharmaceutical advantages, and derivatives with improved pharmacokinetics have been developed [38,39]. In the absence of convulsant, an 18 h exposure to 1.25–5 µM plinabulin lowered the normal swimming behavior of zebrafish larvae, which was not observed at 10 µM. It is unclear what causes this effect, as no visual signs of toxicity or locomotor impairment were observed. From the literature [72], it is known that antiseizure compounds can have locomotor effects on zebrafish larvae without any obvious translation to adverse effects in the rodent model or clinic.

In the mouse 6 Hz psychomotor seizure model, 10–40 mg/kg doses were observed to be comparable in terms of efficacy, and, although significant activity was observed, plinabulin was less effective than the positive control, 300 mg/kg valproate. When valproate is dosed in a suboptimal manner (data not shown), a significant reduction in seizure duration is also observed without complete rescue. The low doses used for plinabulin could also be suboptimal, but no higher doses were tested due to solubility limitations. Based upon our findings in zebrafish, we speculate that in the mouse 6 Hz seizure model, a longer treatment period and repetitive dosing could elevate plinabulin’s uptake and efficacy. Pharmacokinetic analysis would be essential to decide upon the optimal treatment period and dosing scheme. In an antitumor study of Singh and colleagues, mice were treated twice per week for three weeks [47].

As plinabulin is currently in phase III clinical trials, it could have potential for drug repurposing towards epilepsy. Plinabulin is a microtubule destabilizing agent, exerting its function via reversible binding to β-tubulin within the colchicine pocket, thereby inhibiting tubulin polymerization and blocking cell division. More specifically, β-tubulin plays a crucial role in the formation of the components of cellular mitosis, such as the cytoskeleton. Since cancer cells show a higher rate of mitosis, their vulnerability to the actions of plinabulin increase, partially clarifying its mechanism of action [58,73,74]. The lack of antiseizure activity of the functional analogues, colchicine and indibulin, suggests that microtubule depolymerization is not likely to be involved in its antiseizure mode of action. In addition to this tumor cytotoxicity mechanism, another mode of action reported for plinabulin is the guanine nucleotide exchange factor-H1 (GEF-H1), typically released upon microtubule stabilization and required for dendritic cell activation. The release of GEF-H1 results in the activation of an innate immune-signaling pathway, initiating dendritic cell (DC) maturation and differentiation, as well as T-cell activation. Thus, the binding of plinabulin and subsequent activation of the GEF-H1 signaling pathway enhances the presentation of tumor antigens to CD8 T-cells, resulting in antitumor immunity [75]. While other colchicine-site microtubule destabilizing agents, such as colchicine, ansamitocin-P3, and vinblastine, have a similar capacity to induce DC maturation and T-cell dependent tumor control, the signaling pathways in DCs, acting downstream of microtubule destabilization, remain unclear [75]. Surprisingly, microtubule destabilizing agents such as colchicine are reported to increase or cause neutropenia, whereas plinabulin is used as a CIN-therapy [76,77,78]. This highlights the differences of plinabulin in comparison to other agents binding to the same β-tubulin pocket [76]. Indeed, when looking closely at the colchicine binding site of β-tubulin, plinabulin has an altered binding profile, which might explain the changes in anticancer activity [76]. In the case of epilepsy, however, it is not yet clear how plinabulin exerts its antiseizure activity. Most of the recent advances in epilepsy research have focused on the role of synaptic components in the pathogenesis of epilepsy, rather than potential actions of the cytoskeleton. Especially in neurons, the cytoskeleton covers a wide range of pivotal functions such as cell division, intracellular transport, neural proliferation and migration, synaptic transmission, neurotrophic support, voltage-gated ion channel modulation, et cetera [79,80]. Despite the fact that alterations in any of these events may lead to an impaired cytoskeletal network and, consequently, the development of neurodevelopmental disorders and epileptic syndromes, the question remains as to how the actions of the microtubule destabilizer plinabulin can produce antiseizure and anti-epileptiform effects, while the other agents do not. A potential hypothesis is the role of innate immune system activation in the pathogenesis of epilepsy. However, the function of DC and T-cell activation in epileptogenesis and seizure control has been largely unexplored [81]. One could suggest that, as already cited above, the alternative activation profile of the GEF-H1 release among microtubule destabilizing agents could induce different DC and T-cell maturation effects and thus downstream immune effects, thereby promoting seizure control [75].

Clearly, there are currently more questions than answers when it comes to the mode of action underlying plinabulin’s antiseizure action. Understanding its molecular mechanisms and how it differs from its known applications in the cancer field will be key to evaluating whether its therapeutic use could be broadened. Safety is a main concern in this regard, as ASDs are given long-term and should present no or minimal adverse effects to be favorable. Microtubule destabilizers are associated with relatively severe side effects. Colchicine, for example, has been reported to induce gastrointestinal problems and overdosing can have a poor outcome [76,82]. Other drugs in use, such as combretastatin-A4, are known to generate cardiovascular side effects [76,83]. The adverse-effect profile of plinabulin, on the other hand, is mainly gastrointestinal and much less severe than that observed with colchicine. The observed side effects for microtubule destabilizing agents are primarily declarable by the residence time, i.e., the time during which a ligand is bound to its target. In a recent study published by La Sala et al. [76], it was demonstrated that, for colchicine binding, a long residence time was associated with gastrointestinal toxicity, while a short residence time was associated with cardiotoxicity. Moreover, they propose plinabulin as an ideal compound with a good efficacy and acceptable toxicity profile, achieved by a favorable residence time and, thus, a gastrointestinal-cardiac toxicity profile midway between colchicine and combretastatin-A4. Besides, whereas colchicine and combretastatin-A4 are known to cause or increase CIN, plinabulin is used in patients as an anti-CIN therapy, highlighting its great potential in the clinic [76,77]. Of course, when the anti-epileptic targets of plinabulin are unraveled, hit-to-lead optimization could identify specific compounds that do not bind to β-tubulin.

## 4. Materials and Methods

### 4.1. Chemical Experimental Procedures

UHPLC-DAD-QTOFMS was performed on an Agilent Infinity 1290 UHPLC system (Agilent Technologies, Santa Clara, CA, USA) equipped with a diode array detector. Separation was achieved on an Agilent Poroshell 120 phenyl-hexyl column (2.1 × 150 mm, 2.7 µm) with a flow of 0.35 mL/min at 60 °C using a linear gradient 10% acetonitrile (MeCN) in Milli-Q water buffered with 20 mM formic acid (FA) increased to 100% in 15 min staying there for 2 min, returned to 10% in 0.1 min and kept there for 3 min before the following run. MeCN was LC-MS grade. MS detection was conducted using an Agilent 6550 iFunnel QTOFMS equipped with an Agilent Dual Jet Stream electrospray ion source with a drying gas temperature of 160 °C, gas flow of 13 L/min, sheath gas temperature of 300 °C, and flow of 16 L/min. The capillary voltage was set to 4000 V and the nozzle voltage to 500 V. Data processing was performed using Agilent MassHunter Qualitative Analysis for quadrupole time of flight (v.B.07.00). The elemental composition of peaks corresponding to halimide were identified based on mass accuracy and isotopic ratios, as well as the isotopic pattern. Pre-fractionations were performed using flash chromatography of the crude extracts with an Isolera One automated flash system (Biotage, Uppsala, Sweden). Purification of the scalemic mixture and the individual enantiomers of halimide was performed using a Waters 600 Controller (Milford, MA, USA) coupled to a Waters 996 Photodiode Array Detector. The 1D and 2D NMR experiments were conducted using standard pulse sequences on a 600 MHz Bruker Ascend spectrometer with a SmartProbe (BBO). Optical rotations were measured on a Perkin Elmer 341 polarimeter (Perkin Elmer, Waltham, MA, USA).

### 4.2. Microbial Strain and Microbial Cultivation

*Aspergillus insuetus* IBT 28443 was sourced from the IBT culture collection at the Department of Biotechnology and Biomedicine, Technical University of Denmark. The fungus was isolated from a seawater trap set in the North Sea, in between Denmark and Norway, and cultivated in small scale as previously described [3]. For cultivation on a large scale, the fungus was cultivated on 250 plates of CYA for 9 days in the dark at 25 °C.

*Aspergillus ustus* IBT 4133 was sourced from the IBT culture collection at the Department of Biotechnology and Biomedicine, Technical University of Denmark. For the cultivation in large scale the fungus was cultivated on 140 CYA media plates for 7 days in the dark at 25 °C.

### 4.3. Microbial Extraction and Isolation

The extraction and fractionation of the small-scale cultivations of *Aspergillus insuetus* IBT 28443 (both combined (on CYA and YES) and individual (on CYA, YES and Oatmeal agar)) was performed as previously described [3]. The large-scale cultivation of *Aspergillus insuetus* IBT 28443 (on CYA) was extracted with 150 mL ethyl acetate (EtOAc), with 1% FA for every 10 plates. The EtOAc crude extract was fractionated on a reversed-phase C_18_ flash column (Sepra ZT, Isolute, 25 g/33 mL) using the Isolera One automated flash system. The gradient was 10% stepwise (12 column volumes (CV)) from 15% to 100% MeCN buffered with 20 mM FA and using a flow of 25 mL/min. Fractions were collected manually for every 10%. The most bioactive fraction, SK1312, 25% MeCN, was fractionated on a reversed phase Isolute SPE column (500 mg/3 mL) using methanol (MeOH) buffered with 20 mM FA. The metabolites were eluted with 2 CV per fraction: 15% MeOH, 20% MeOH, 30% MeOH, 40% MeOH, 50% MeOH, 60% MeOH, 80% MeOH, and 100% MeOH. From the 50% MeOH, 60% MeOH (SK1414), and 80% MeOH (SK1415) isolera fractions, halimide separation was achieved on a Gemini C_6_ Phenyl, 5 μm, 250 × 10 mm column (Phenomenex, Torrance, CA, USA) with a flow of 4 mL/min. A linear gradient of 40% MeCN in Milli-Q water buffered with 20 mM FA going to 70% MeCN in 30 min was used.

For the large-scale cultivation of *Aspergillus ustus* IBT 4133, the 140 plates were extracted in seven 1L beakers with 300 mL EtOAc per 20 plates. The EtOAc crude extract was fractionated on a reversed-phase C_18_ flash column (15 µm/100 Å, 25 g/33 mL) using the Isolera One automated flash system. MeCN and Milli-Q water were buffered with 20 mM FA and the flow was 25 mL/min. The gradient was stepwise from 15% to 100% MeCN and metabolites were eluted with CV per fraction: 12 CV 15% MeCN, 6 CV 22% MeCN, 12 CV 25% MeCN, 6 CV 27% MeCN, 12 CV 30% MeCN, 12 CV 35% MeCN, 12 CV 65% MeCN, and 12 CV 100% MeCN. Halimide purification was achieved from the 25% MeCN fraction on a Kinetex C_18_, 5 μm, 250 × 10 mm column (Phenomenex, Torrance, CA, USA) with a flow of 4 mL/min. A linear gradient of 25% MeCN in Milli-Q water buffered with 20 mM FA going to 75% MeCN in 30 min was used.

Separation of the *R*- and *S*-enantiomers of halimide was achieved on a Lux Cellulose-1, 3 μm, 100 × 4.6 mm column (Phenomenex, Torrance, CA, USA) with a flow of 2 mL/min and using a linear gradient of 20% MeCN in Milli-Q water going to 80% MeCN in 20 min.

Halimide (scalemic mixture): yellow solid; ${\left[\alpha \right]}_{D\;}^{20}$ +78 (*c* 0.24, MeOH); UV (MeCN) λmax: 205 nm; 236 sh nm; 320 nm; ^1^H- and ^13^C-NMR (Table 1, Figures S4 and S5) and HRESIMS *m/z* 351.1818 [M+H]^+^ (calculated for C_20_H_23_N_4_O_2_, *m/z* 351.1816, ∆ −0.77); *R*-enantiomer: ${\left[\alpha \right]}_{D\;}^{20}$ +213 (*c* 0.27, MeOH); and *S*-enantiomer: ${\left[\alpha \right]}_{D\;}^{20}$ −200 (*c* 0.09, MeOH).

#### Marfey’s Analysis

A total of 50 µg of halimide was hydrolyzed in 6 M hydrogen chloride (HCl) at 110 °C for 24 h. After hydrolysis the sample was dried using N_2_ steam. A total of 100 µL 0.125 M borate buffer and 100 µL 1% 1-fluoro-2-4-dinitrophenyl-5-L-alanine amide (FDAA) in acetone was added to the hydrolysis product or L- and D-phenylalanine (2.5 µmol). This reaction was heated to 40 °C for 1 h. The reaction was quenched by addition of 20 µL 1 M HCl and, prior to UHPLC-DAD-QTOFMS analysis, 400 µL MeOH was added to the solution.

### 4.4. Compounds

**Figure 11:**
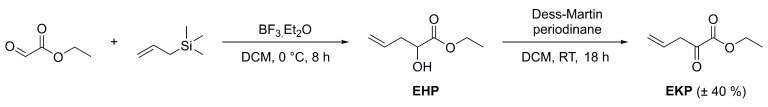
Synthesis of ethyl ketopentenoate (EKP) via Lewis acid-catalyzed allylation of ethyl glyoxylate followed by Dess-Martin oxidation. Abbreviations: dichloromethane: DCM; ethyl hydroxypentenoate: EHP; 3-oxo-1λ^5^-benzo[*d*][1,2]iodaoxole-1,1,1(3*H*)-triyl triacetate: Dess-Martin periodinane; and room temperature: RT.

Plinabulin (>98% purity) was purchased at Adooq BioScience (Irvine, CA, USA), PTZ (≥99% purity) and valproate (sodium valproate, ≥98% purity) were purchased from Sigma-Aldrich (Overijse, Belgium), perampanel (>98% purity) and colchicine (>98% purity) from Bio-Connect (Huissen, The Netherlands), and indibulin (≥98% purity) from Tocris Bioscience (Abingdon, UK). EKP was synthesized in several batches using an in-house-optimized literature procedure [27] (Figure 11).

### 4.5. Compound and Sample Preparation

For experiments using zebrafish larvae, dry samples and compounds were dissolved in 100% dimethyl sulfoxide (DMSO, spectroscopy grade, Acros Organics (Geel, Belgium)) as 100-fold-concentrated stocks and diluted in embryo medium to a final concentration of 1% DMSO content, except for PTZ which was dissolved in embryo medium (0% DMSO). Control groups were treated with 1% DMSO (VHC) in accordance with the final solvent concentration of tested samples or compounds. For mice experiments, a mixture of poly-ethylene glycol M.W. 200 (PEG200, >95% purity, Acros Organics (Geel, Belgium)) and DMSO (1:1 PEG200:DMSO) was used as solvent and VHC.

### 4.6. Experimental Animals

All animal experiments carried out were approved by the Ethics Committee of the University of Leuven (approval numbers 101/2010 (zebrafish), 061/2013 (mouse), 150/2015 (zebrafish), 023/2017 (zebrafish), and 027/2017 (mouse)) and by the Belgian Federal Department of Public Health, Food Safety and Environment (approval numbers LA1210199 and LA1210261) in accordance with the EU Directive 2010/63/EU.

#### 4.6.1. Zebrafish

Adult zebrafish (*Danio rerio*) stocks of AB strain (Zebrafish International Resource Center, Eugene, OR, USA) were maintained at 28 °C on a 14/10 h light/dark cycle under standard aquaculture conditions. Fertilized eggs were collected via natural spawning and raised in embryo medium (1.5 mM HEPES, pH 7.2, 17.4 mM NaCl, 0.21 mM KCl, 0.12 mM MgSO_4_, 0.18 mM Ca(NO_3_)_2_, and 0.6 μM methylene blue) at 28 °C under constant light with regards to the zebrafish PTZ seizure model and under a 14/10 h light/dark cycle with regards to the zebrafish EKP seizure model.

#### 4.6.2. Mice

Male NMRI mice (weight 18–20 g) were acquired from Charles River Laboratories (Écully, France) and housed in polyacrylic cages under a 14/10 h light/dark cycle at 21 °C. The animals were fed a pellet diet and water ad libitum and were allowed to acclimate for one week before experimental procedures were conducted. Prior to the experiment, the mice were isolated in polyacrylic cages with a pellet diet and water ad libitum for habituation overnight in the experimental room to minimize stress.

### 4.7. Toxicity Evaluation

The MTC was determined as described previously [84], prior to further experiments, and used as the highest test concentration. In brief, 12 larvae of 6 or 7 dpf were individually exposed to a certain concentration within a concentration range (2-fold dilution series), in a 100 µL volume for 18 h in a 96-well plate at 28 °C in the dark. The following parameters were investigated after 2 and 18 h of exposure: touch response, morphology, posture, edema, signs of necrosis, swim bladder, and heartbeat. The MTC was defined as the highest concentration at which no larvae died nor showed signs of toxicity or locomotor impairment in comparison to VHC-treated control larvae. In case no MTC was reached, the highest soluble concentration was used.

### 4.8. Behavioral Analysis

Experiments were performed as described previously [2,35]. 7 dpf larva (in the case of 2 h incubation) or 6 dpf larva (in the case of 18 h incubation) were individually treated with either VHC (1% DMSO) or the test compound (1% DMSO) in a 100 µL volume for 2 or 18 h in a 96-well plate at 28 °C in the dark. Then, 100 µL of either VHC (0 or 1% DMSO), 40 mM PTZ (0% DMSO, 20 mM working concentration), or 600 µM EKP (1% DMSO, 300 µM working concentration) was added to each well. Next, within 5 min, the 96-well plate was placed in an automated tracking device (ZebraBox, ViewPoint, Lyon, France), and larval behavior was video recorded for 30 min. The complete procedure was performed in the dark using infrared light. Total locomotor activity was recorded by ZebraLab software (ViewPoint, Lyon, France) and expressed in actinteg units, which is the sum of pixel changes detected during the defined time interval (5 min). Larval behavior was depicted as mean actinteg units per 5 min, during the 30 min recording period (PTZ model) or during the 10–25 min recording period (EKP model), and over consecutive time intervals. Data are expressed as mean ± SEM.

### 4.9. Electrophysiology

Non-invasive LFP recordings were measured from the midbrain (optic tectum) of 7 dpf zebrafish larvae pre-incubated with VHC only, PTZ or EKP only, compound and VHC, or compound and PTZ or EKP, as described previously [2,27,35]. Larvae were treated as described above. After incubation, an equal volume of either VHC (0 or 1% DMSO), 40 mM PTZ (0% DMSO, 20 mM working concentration), or 600 µM EKP (1% DMSO, 300 µM working concentration) was added to the well for 15 min prior to the recording. These steps occurred at 28 °C, while further manipulation and electrophysiological recordings occurred at room temperature (21 °C). The larva was embedded in 2% low-melting-point agarose (Invitrogen, Carlsbad, CA, USA), and the signal electrode (an electrode inside a blunt soda-glass pipet (1412227, Hilgenberg, Germany) was pulled with a DMZ Universal Puller (Zeitz, Germany), diameter ± 20 microns, containing artificial cerebrospinal fluid (ACSF: 124 mM NaCl, 10 mM glucose, 2 mM KCl, 2 mM MgSO_4_, 2 mM CaCl_2_, 1.25 mM KH_2_PO_4_, and 26 mM NaHCO_3_, 300–310 mOsmols)), which was positioned on the skin covering the optic tectum. A differential extracellular amplifier (DAGAN 2400 amplifier, Minneapolis, MN, USA) amplified the voltage difference between the signal (measured by the signal electrode) and the reference electrode. The differential signal was band-pass filtered at 0.3–300 Hz and digitized at 2 kHz via a PCI-6251 interface (National Instruments Belgium NV, Zaventem, Belgium) using WinEDR (John Dempster, University of Strathclyde, Glasgow, UK). A grounding electrode grounded the electrical system. All electrodes were connected with ACSF. Each recording lasted 600 s. Automated power spectral density analysis was performed using Matlab R2019b and R2021b (The MathWorks, Inc., Natick, MA, USA), as described previously [35], using specifically developed software [85]. Electrophysiological data was normalized against the VHC + VHC control condition and expressed as mean ± SEM per larva within the 20–90 Hz region and as mean ± SEM per larva per 10 Hz frequency band from 1–150 Hz.

### 4.10. Mouse 6 Hz (44 mA) Psychomotor Seizure Model

Experiments were performed as previously described [2]. In brief, 50 µL (injection volume was adjusted to the individual weight) of VHC (PEG200:DMSO 1:1) or treatment (valproate or plinabulin dissolved in VHC) was i.p. injected in NMRI mice (average weight 32 g, range 28–36 g), and, after 30 min, psychomotor seizures were induced by corneal electrical stimulation (6 Hz, 0.2 ms rectangular pulse width, 3 s duration, 44 mA) using an ECT Unit 5780 (Ugo Basile, Comerio, Italy). Seizure durations were measured during the experiment by experienced researchers who were familiar with the different seizure behaviors. In addition, seizure durations were determined by blinded video analysis to confirm or correct the initial observations. Data are expressed as mean ± SD.

### 4.11. Statistical Analysis

Statistical analyses were performed using either one-way ANOVA (more than two groups and one variable) or two-way ANOVA (more than two groups and two variables) with Dunnett’s multiple comparison test in GraphPad Prism 9 (San Diego, CA, USA). Data are expressed as mean ± SEM or mean ± SD, as indicated. Animals were randomly allocated to experimental groups.

## 5. Conclusions

In conclusion, this study shows for the first time that 2,5-diketopiperazines have antiseizure properties, and that the clinical drug candidate plinabulin is active against drug-resistant epileptic seizures in zebrafish and mice. Although the antiseizure mode of action is currently unclear, the inactivity of functional analogues suggest that it is unrelated to microtubule depolymerization. Further research will be crucial to understand the therapeutic value of these findings, and to investigate whether the medical use of plinabulin might be broadened from the cancer field to include epilepsy.

## 6. Patents

PCT patent publication WO2019043012 (PCT/EP2018/073147), Copmans D., Crawford A., de Witte P., Esguerra C., Kildgaard S., Ostenfeld Larsen T., Ny A. Treatment of epilepsy with plinabulin or halimide or diketopiperazine derivatives, Katholieke Universiteit Leuven and Technical University of Denmark.

## Figures and Tables

**Table 1:** ^1^H- and ^13^C-NMR spectroscopic data of halimide (scalemic mixture).

	Halimide	
Position	δ^1^H (Mult, J)	δ^13^C
1	-	-
2	7.67 s	135.1
3	-	-
4	-	132.4
5	-	138.7
6	6.60 s	107.2
7	-	124.4
8	-	-
9	-	167.4
10	4.46 t(4.4)	58.1
11	-	-
12	-	162.3
13	3.06 dd(13.6,4.4)	41.4
	3.27 dd(13.6,4.4)	
14	-	135.9
15/19	7.18 m	131.5
16/18	7.20 m	129.6
17	7.15 m	128.4
20	-	38.8
21	5.99 dd(17.5,10.6)	146.6
22	5.02 dd(17.5,1.0)	113.1
	5.08 dd(10.6,1.0)	
23/24	1.41 d(6.6)	28.6

NMR spectroscopic data (600 MHz, MeOD, δ in ppm, *J* in Hz) for halimide isolated from the EtOAc crude extract of *Aspergillus insuetus* IBT 28443.

## Data Availability

The data supporting the reported results are digitally archived and can be obtained from the corresponding authors upon reasonable request.

## Supplementary Materials

The following are available online at https://www.mdpi.com/article/10.3390/ph15020247/s1: Figure S1: Behavioral and electrophysiological antiseizure analysis of positive controls valproate and perampanel in the zebrafish PTZ and EKP seizure model, respectively. Figure S2: Representative local field potential recordings of the zebrafish PTZ seizure model. Figure S3: Representative local field potential recordings of the zebrafish EKP seizure model. Figure S4: ^1^H-NMR (600 MHz, MeOD) of halimide. Figure S5: ^13^C-NMR (150 MHz, MeOD) of halimide.

## Abbreviations

Antiseizure drug: ASD; base peak chromatogram: BPC; chemotherapy-induced neutropenia: CIN; czapek yeast extract agar: CYA; days post-fertilization: dpf; ethyl ketopentenoate: EKP; γ-aminobutyric acid: GABA; glutamic acid decarboxylase: GAD; guanine nucleotide exchange factor-H1: GEF-H1; hours post-fertilization: hpf; local field potential: LFP; marine natural product: MNP; maximum tolerated concentration: MTC; non-small cell lung cancer: NSCLC; pentylenetetrazole: PTZ; photomotor response: PMR; positive electrospray ionization mode: ESI+; power spectral density: PSD; ultra-high performance liquid chromatography-diode array detection-quadrupole time of flight mass spectrometry: UHPLC-DAD-QTOFMS; vehicle: VHC; yeast extract sucrose agar: YES.
