# Supplementary material for: From the North Sea to Drug Repurposing, the Antiseizure Activity of Halimide and Plinabulin
Source: Pharmaceuticals (Basel). 2022 Feb 18;15(2):247. doi: 10.3390/ph15020247 (PMC8878679; doi:10.3390/ph15020247)
Supplement: Supplementary file 1 [file pharmaceuticals-15-00247-s001.zip › pharmaceuticals-1553696-supplementary.pdf]

Supporting information

From the North Sea to Drug Repurposing, the Antiseizure Activity of Halimide and Plinabulin

Daniëlle Copmans [1,*,†], Sara Kildgaard [2,†], Emma Roux [1], Michèle Partoens [1], Gert Steurs [3], Xinhui Wang [2], Wim M. De Borggraeve [3], Camila V. Esguerra [1,4], Alexander D. Crawford [1,5], Thomas O. Larsen [2,*] and Peter A. M. de Witte [1,*]

[1] Laboratory for Molecular Biodiscovery, Department of Pharmaceutical and Pharmacological Sciences, KU Leuven, Herestraat 49 box 824, 3000 Leuven, Belgium; emma.roux@kuleuven.be (E.R.); michele.partoens@kuleuven.be (M.P.); c.v.esguerra@farmasi.uio.no (C.V.E.); alexander.dettmar.crawford@nmbu.no (A.D.C.)

[2] Department of Biotechnology and Biomedicine, Technical University of Denmark, Søltofts Plads, Building 221, 2800 Kgs. Lyngby, Denmark; sarakildgaard@gmail.com (S.K.); xinwang@dtu.dk (X.W.)

[3] Molecular Design and Synthesis, Department of Chemistry, KU Leuven, Celestijnenlaan 200f box 2404, 3001 Leuven, Belgium; gert.steurs@kuleuven.be (G.S.); wim.deborggraeve@kuleuven.be (W.M.D.B.)

[4] Current affiliation: Centre for Molecular Medicine Norway, Faculty of Medicine, University of Oslo, Gaustadalléen 21, 0349 Oslo, Norway

[5] Current affiliation: Department of Preclinical Sciences and Pathology, Norwegian University of Life Sciences (NMBU), Elizabeth Stephansens vei 15, 1430 Ås, Norway

* Correspondence: danielle.copmans@kuleuven.be (D.C.); tol@bio.dtu.dk (T.O.L.); peter.dewitte@kuleuven.be (P.A.M.d.W.); Tel.: +3216-33-04-20 (D.C.); +45-4525-2632 (T.O.L.); +3216-32-34-32 (P.A.M.d.W.)

† These authors share first authorship.

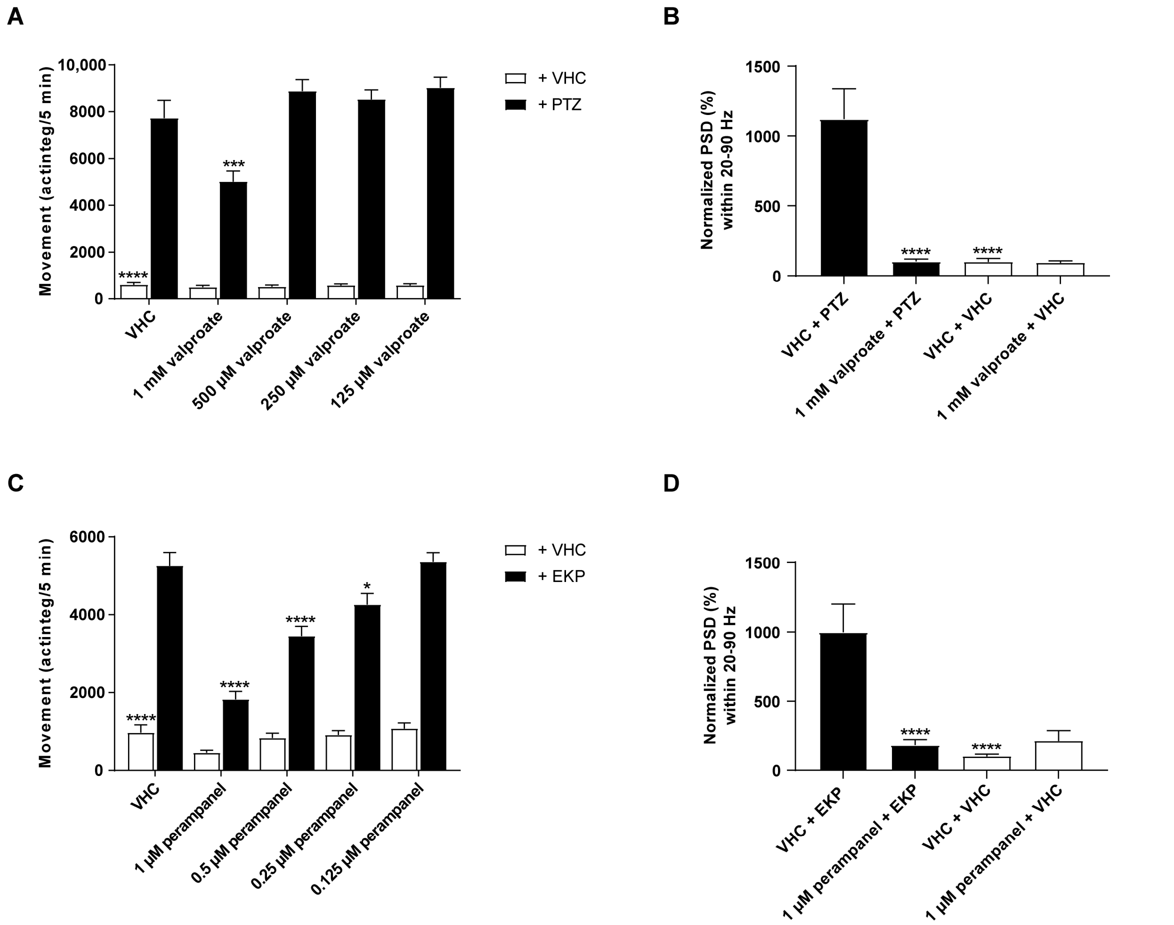

Figure S1: Behavioral and electrophysiological antiseizure analysis of positive controls valproate and perampanel in the zebrafish PTZ and EKP seizure model, respectively. (**A**, **C**) Antiseizure activity of valproate and perampanel in the zebrafish pentylenetetrazole (PTZ) and ethyl ketopentenoate (EKP) seizure model, respectively, after a treatment period of 18 h in case of valproate and 2 h in case of perampanel. Behavioral data is expressed as mean actinteg units per 5 min (±SEM) during the 30 min recording period (A) and during the 10–25 min recording period (**C**). (**B**, **D**) Antiepileptiform activity of valproate and perampanel in the zebrafish PTZ and EKP seizure model, respectively, after a treatment period of 18 h in case of valproate and 2 h in case of perampanel. Electrophysiological data is normalized against the vehicle (VHC) + VHC control condition and expressed as normalized power spectral density (PSD) (mean ±SEM) per larva within the 20–90 Hz region. (**A**, **C**) Data were pooled from three independent experiments with 8–10 replicate wells per test condition (n = 28–30). (**B**, **D**) Number of replicates per test condition were n = 14–17. (**A**-**D**) Statistical analysis: one-way ANOVA with Dunnett's multiple comparison test (GraphPad Prism 9, San Diego, CA, USA). Significance levels: * $p \leq 0.05$; ** $p \leq 0.01$; *** $p \leq 0.001$; **** $p \leq 0.0001$.

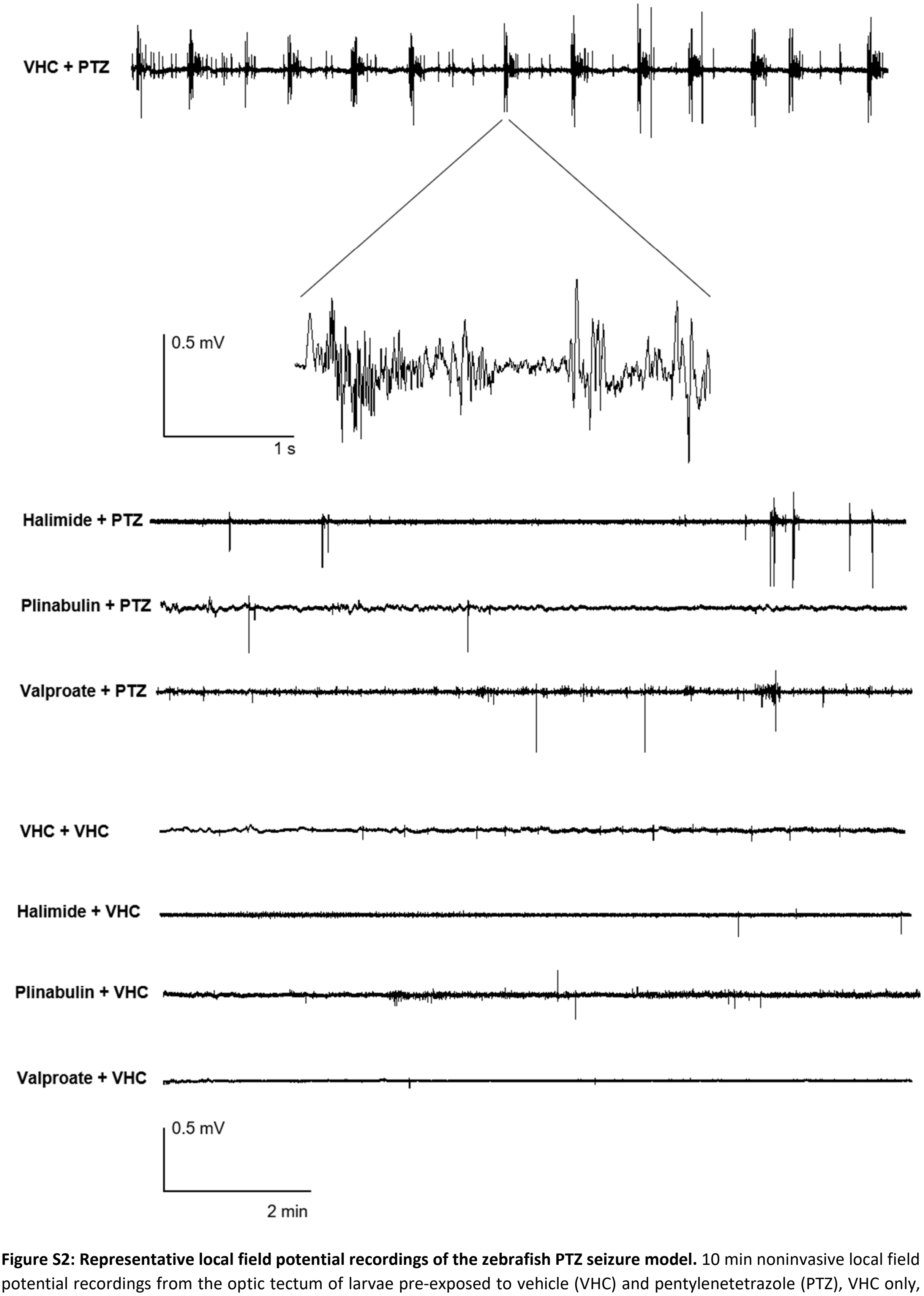

Figure S2: Representative local field potential recordings of the zebrafish PTZ seizure model. 10 min noninvasive local field potential recordings from the optic tectum of larvae pre-exposed to vehicle (VHC) and pentylenetetrazole (PTZ), VHC only, compound and PTZ, or compound and VHC. Test concentration and treatment period used for each compound: 200 µg/mL halimide (2 h), 10 µM plinabulin (18 h), and 1 mM valproate (18 h).

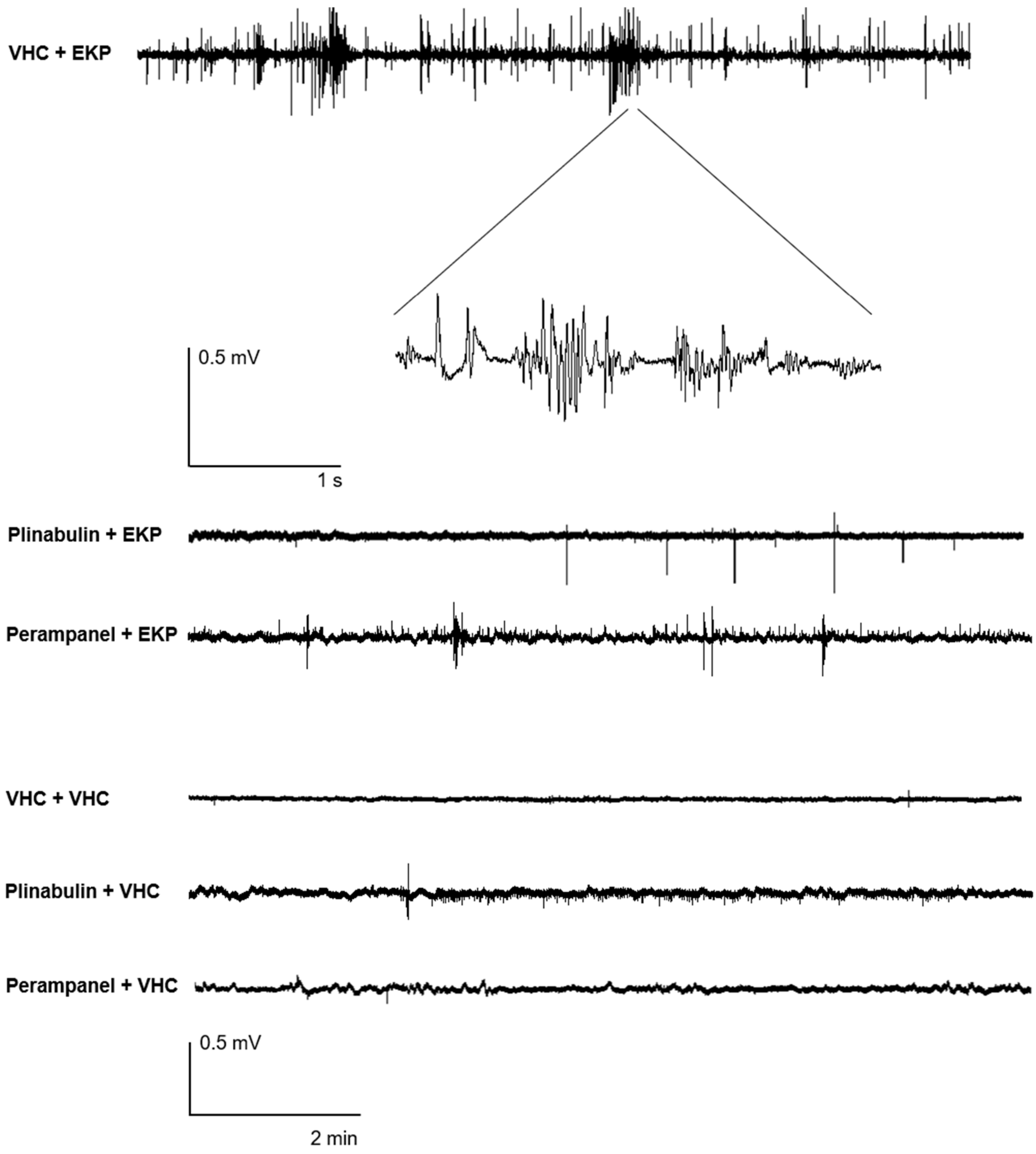

Figure S3: Representative local field potential recordings of the zebrafish EKP seizure model. 10 min noninvasive local field potential recordings from the optic tectum of larvae pre-exposed to vehicle (VHC) and ethyl ketopentenoate (EKP), VHC only, compound and EKP, or compound and VHC. Test concentration and treatment period used for each compound: 10 µM plinabulin (18 h) and 1 µM perampanel (2 h).

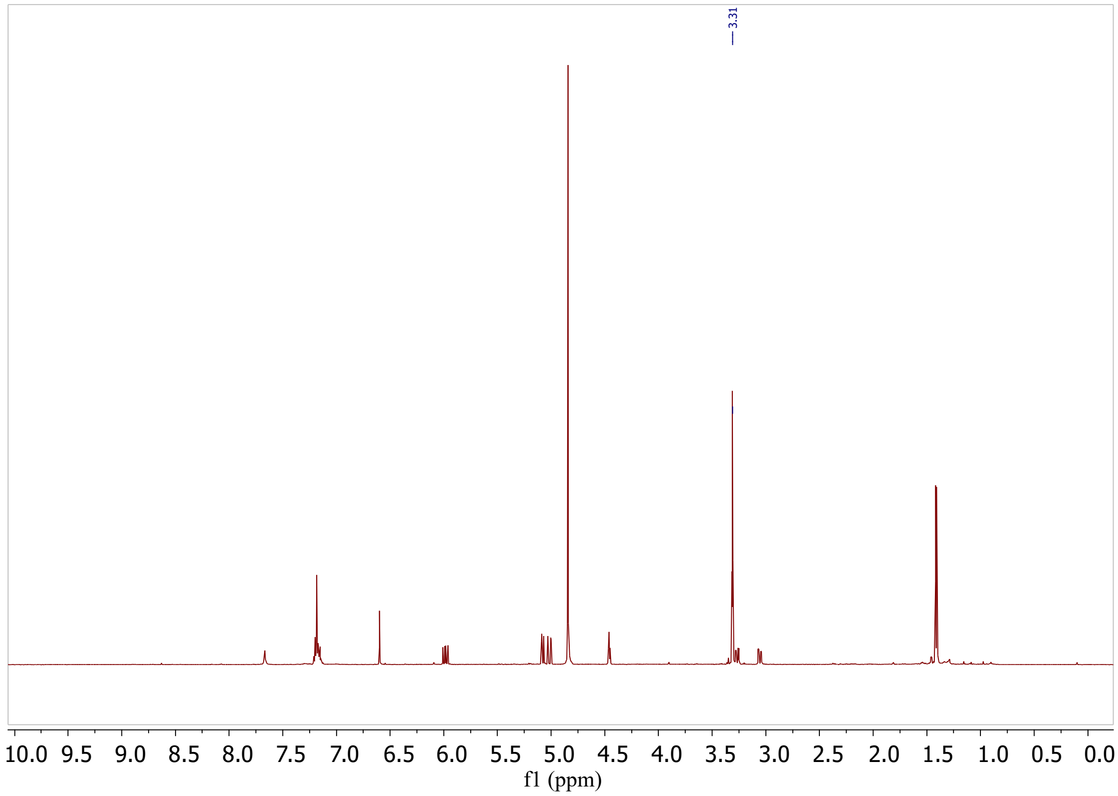

Figure S4: ^{1}H-NMR (600 MHz, MeOD) of halimide.

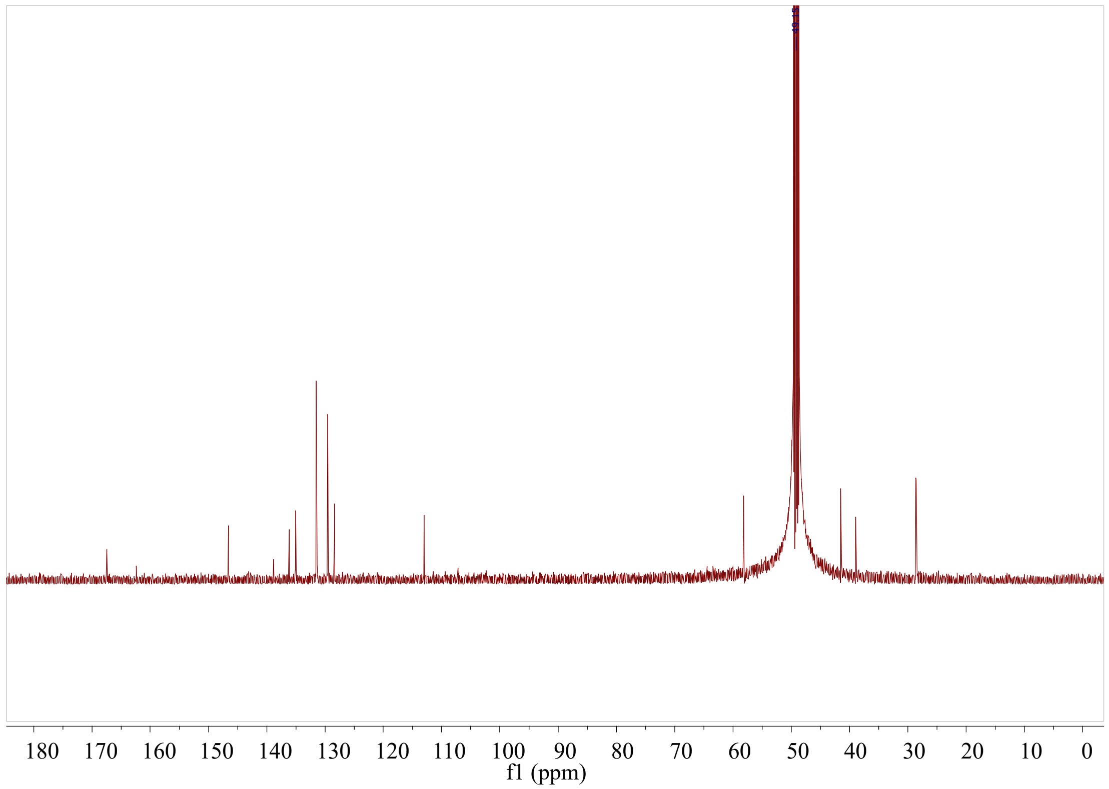

Figure S5: ^{13}C-NMR (150 MHz, MeOD) of halimide.
